# Involvement of MiRNA-211-5p and Arhgap11a Interaction During Osteogenic Differentiation of MC3T3-E1 Cells

**DOI:** 10.3389/fsurg.2022.857170

**Published:** 2022-04-15

**Authors:** Wenwen Ju, Guangfeng Zhang, Xu Zhang, Jingting Wang, Tong Wu, Huafeng Li

**Affiliations:** ^1^Department of Endocrinology (I), The Third Affiliated Hospital of Qiqihar Medical University, Qiqihar, China; ^2^Departments of Magnetic Resonance Imaging (MRI), The Third Affiliated Hospital of Qiqihar Medical University, Qiqihar, China; ^3^Department of Endocrinology, Zhongshan City People's Hospital, Zhongshan, China; ^4^Mental & Health College, Qiqihar Medical University, Qiqihar, China

**Keywords:** osteogenic differentiation, MC3T3-E1 cell, microRNA, miR-211-5p, Arhgap11a

## Abstract

**Objective:**

MicroRNAs (miRNAs) are well-recognized for their abilities to regulate gene expression post-transcriptionally in plants and animals. Recently, miRNA-messenger RNA (mRNA) regulatory relationships have been confirmed during biological processes, including osteogenic differentiation. This study aimed to find out more candidate miRNA-mRNA pairs involved in the osteogenic differentiation of MC3T3-E1 cells.

**Methods:**

An MC3T3-E1-based microarray dataset (accessioned as GSE46400) downloaded from the Gene Expression Omnibus included MC3T3-E1 cells with or without 14-day osteoblast differentiation osteoblast induction. Multiple miRNA-mRNA prediction databases were searched by differentially expressed genes (DEGs) to obtain pairs of a miRNA-DEG regulatory network. The MC3T3-E1 cells were cultured and incubated in the osteogenic differentiation medium for 14 days. The expressions of candidate miRNAs and mRNAs were determined by real-time quantitative PCR(RT-qPCR) in MC3T3-E1 cells. The miRNA-mRNA interactions were verified by dual-luciferase reporter gene assays and experiments using mimics miRNA or their inhibitors.

**Results:**

We identified 715 upregulated DEGs and 603 downregulated DEGs between MC3T3-E1 cells with and without osteoblast induction by analyzing the raw data of the GSE46400 dataset. There were 7 overlapped miRNA-mRNA pairs identified during osteogenic differentiation of MC3T3-E1 cells, including mmu-miR-204-5p-Arhgap11a, mmu-miR-211-5p-Arhgap11a, mmu-miR-24-3p-H2afx, mmu-miR-3470b-Chek2, mmu-miR-3470b-Dlgap5, mmu-miR-466b-3p-Chek1, and mmu-miR-466c-3p-Chek1. The Arhgap11a, H2afx, Chek2, Dlgap5, and Chek1 were hub genes downregulated in MC3T3-E1 cells after osteogenic differentiation, verified by RT-qPCR results. The RT-qPCR also determined declined expressions of miR-204-5p and miR-24-3p concomitant with elevated expressions of miR-211-5p, miR-3470b, miR-466b-3p, and miR-466c-3p in the MC3T3-E1 cells, with osteoblast induction compared with undifferentiated MC3T3-E1 cells. Dual-luciferase reporter gene assays demonstrated Arhgap11a as the target of miR-211-5p. MiR-211-5p upregulation by its mimic increased Arhgap11a expression in MC3T3-E1 cells.

**Conclusion:**

Our study characterizes miR-211-5p targeting Arhgap11a promotes the osteogenic differentiation of MC3T3-E1 cells, which provides novel targets to promote the osteogenesis process during bone repair.

## Introduction

Osteoporosis is characterized by fragile bones, reduced bone mass, and bone microstructure deterioration (1). It has been reported that 1.3 million suffered from fractures due to osteoporosis annually (2), and estimated that men and women over the age of 50 had osteoporosis-related fractures, accounting for 50 and 20%, respectively (3). The impairment of proliferation, differentiation, and mineralization in osteoblast is the main pathophysiological mechanism of osteoporosis, which has been proven by previous studies (4, 5). Osteoblast mainly originates from mesenchymal stem cells (MSCs), and it is involved in bone formation, remodeling, and reconstruction *via* the formation of a bone matrix and regulation of bone resorption (6). Alkaline phosphatase (ALP), osteocalcin (OCN), osteopontin (OPN), and runt-related transcription factor 2 (Runx2) are commonly used to evaluate differentiation and maturation of osteoblasts. Runx2 belongs to the Run family and is an essential upstream transcription factor during osteoblast differentiation. It regulates Sp7 protein expression and induces expression of major bone matrix protein genes, such as Col1a1, Spp1, Ibsp, Bglap2, and Fn1 (7, 8). Osterix (OSX) is a transcription factor in the SP family, which is essential for osteoblast differentiation, and its expression is closely related to Runx2 (9). The ALP is a marker widely used in early osteoblast differentiation, and its significance has been identified in the diagnosis and treatment of metabolic bone diseases (10). The ALP increases the local rate of inorganic phosphate and promotes mineralization. High activity of ALP is found in the cells of mineralized tissues (11). The zOCN, a rich protein produced by osteoblasts, is mainly responsible for bone mineralization, synthesis, and deposition. It is a serum marker of osteoblast bone formation and regulates mineralization in a bone matrix (12). Its extensive role in the regulation of systemic metabolism, reproduction, and cognition has been confirmed (13). In 1985, Franzén et al. isolated OPN protein from a bovine bone (14). As a marker of osteoblasts, OPN is involved in cell communication and matrix mineralization in bone regeneration and development (15). The OPN expression was found in various cells, including osteoblasts (16), osteocytes (17), and bone marrow's MSCs (18).

Relevant pieces of research have shown that the osteogenic differentiation of stem cells is affected by microRNAs (miRNAs) (19). The miRNAs are involved in cells proliferation, differentiation, survival, and apoptosis through targeting specific mRNA (20). The study indicated that low expression of SMAD4 targeted by miR-224 hindered osteoblast differentiation (21). Another data showed that mir-130a accelerated osteoblast differentiation by negatively regulating the expression of Smurf2 in bone marrow MSCs (22). The MC3T3-E1 mouse pre-osteoblast cell line is a classic model for studying the process of osteogenic differentiation *in vitro* due to its high proliferation and differentiation properties. The MC3T3-E1 cells formed mineralized nodules *in vitro* in the presence of ascorbic acid (23). Therefore, the present study focuses on candidate miRNA-mRNA pairs involved in osteogenic differentiation of MC3T3-E1 cells to provide novel targets to facilitate bone repair in treating skeletal diseases.

## Methods

### The Download of a Microarray Dataset and Differential Expression Analysis

An MC3T3-E1-based microarray dataset (accessioned as GSE46400) was downloaded from the Gene Expression Omnibus (GEO, https://www.ncbi.nlm.nih.gov/gds). The GSE46400 was generated on the GPL6246 platform, including three independent replicates for the MC3T3-E1 cell line undergoing 14-day osteoblast differentiation and three independent replicates for the MC3T3-E1 cell line without osteoblast induction. The osteogenic differentiation medium consisted of dexamethasone, β-glycerophosphate, and L-ascorbic acid. Differentially expressed genes (DEGs) that were upregulated or downregulated more than |log2 fold change (FC)| > 1 (corrected *p* < 0.05) between MC3T3-E1-derived-mature osteoblasts and control MC3T3-E1 were screened by analyzing raw data of the GSE46400 using the affy and limma package from the R/Bioconductor software.

### Functional Enrichment Analysis

The Gene Ontology (GO) database was employed to analyze the function of the DEGs according to GO terms, which is an international standard classification system of the National Center for Biotechnology Information. The GO functional annotation involves three fields: biological process (BP), cellular component (CC), and molecular function (MF), and each field includes a series of gene items. Pathway analysis was performed to find out the most significant pathways enriched by DEGs according to KEGG, which is a public database for systematic analysis of gene functions about the networks of genes and molecules. The DEGs were entered into the GO and KEGG databases to obtain GO terms and KEGG pathways significantly enriched by DEGs.

### miRNA-mRNA Pairs

The DEGs were mapped into multiple miRNA-mRNA prediction databases, including miRTarBase (http://miRTarBase.cuhk.edu.cn/), TargetScan (http://www.targetscan.org/vert_71/), miRDB (http://mirdb.org/), and miRanda (https://www.cs.kent.ac.uk/people/staff/dat/miranda/) to screen out putative miRNA targeting hub genes. Next, we mapped putative miRNA and hub genes using cytoscape3.4.0 software; we obtained pairs of the miRNA-DEG regulatory network.

### Protein-Protein Interaction (PPI) Network Construction

The STRINGonline database (http://string-db.org) was used as the search tool for the retrieval of interacting genes to construct a PPI network, which is undirected graphs with nodes corresponding to proteins and unit-weight edges, reflecting the interactions between two proteins. During ongoing research, DEGs were mapped into the InputBox, indicating multiple proteins. Once the organism was selected, the bottom of the search was clicked. When the results were yielded, the bottom of exports was clicked, with tab separated values (TSV)-typed files downloaded. In addition to the STRING database, the integrated regulatory networks were constructed using the Cytoscape plugin cytoHubba. The Cytoscape is an open-source JavaScript-based graph library for integrating biomolecular interaction networks with high-throughput expression data and other molecular states into a unified conceptual framework. The Cytoscape is most powerful when used in conjunction with large databases of protein-protein, protein-DNA, and genetic interactions that are increasingly available for humans and model organisms. The TSV-typed files downloaded from the STRING database were imported into the Cytoscape, with hub genes obtained. Interactions with a combined score >0.9 and connectivity ≥10 were regarded as significant for hub genes.

### Cell Culture and Osteoblast Induction

The MC3T3-E1 cells (the Cell Bank of the Chinese Academy of Sciences, Shanghai, China) were maintained in the MEM, containing 10% fetal bovine serum (FBS; Thermo Fisher Scientific, USA) and 1% penicillin-streptomycin in a humidified culture chamber with 10% CO_2_ at 37°C. When cell confluence reached 80%, the MC3T3-E1 cells were incubated in the osteogenic differentiation medium, containing 100-nM dexamethasone, 10-mM β-glycerophosphate, and 0.2-mM l-ascorbate (24). A fresh medium was refreshed every second day for 14 days.

### RNA Extraction and Real-Time qPCR (RT-qPCR)

Following total RNA extraction from MC3T3-E1 cells by Trizol methods (Invitrogen, USA), complement DNA (cDNA) was reverse-transcribed using the PrimeScript RT reagent Kit (Takara, Japan) for mRNAs and amplified using the SYBR^®^Premix ExTaqTM II (RR820A, Takara) kit, and the ABI PRISM^®^7300 System (ABI, USA). Data were relative to the expression of the reference gene GADPH and analyzed using the 2^−ΔΔCt^ method. The primers were synthesized by RiboBio (Guangzhou, China), and their sequences are listed in Table 1. Taqman probes were purchased (Invitrogen) for quantification of mmu-miRs: miR-204-5p, miR-211-5p, miR-24-3p, miR-3470b, miR-466b-3p, miR-466c-3p, with snoRNA202 as a loading control. TaqMan Universal PCR Master Mix without AmpErase UNG was employed in accordance with the manufacturer's protocol.

**Table 1:** Primer sequences used for RT-qPCR.

**Target**	**Primer sequences (5^**′**^-3^**′**^)**
Arhgap11a	F: 5′-GCAGGTGTGCCAAGGCGAAGT-3′
	R: 5′-TGCAAGTCGCCAACCAACACTTTCA-3′
H2afx	F: 5′-ACGACGAGGAGCTCAACAAG-3′
	R: 5′-TAGTACTCCTGGGAGGCCTG-3′
Chek2	F: 5′-TTATCTGCCTTAGTGGGTATCCA-3′
	R: 5′-CTGTCGTAAAACGTGCCTTTG-3′
Dlgap5	F: 5′-AAGTGGGTCGTTATAGACCTGA-3′
	R: 5′-TGCTCGAACATCACTCTCGTTAT-3′
Chek1	F: 5′-ACCTGCTTTACATTTCCACTTG-3′
	R: 5′-ACAGCAAACAGAGGAGGTTATT-3′
ALP	F: 5′-AACAGACAAGCAACCCAAAC-3′
	R: 5′-TAACCCAACGGGCAGAAA-3′
OSX	F: 5′-CAAATACCC AGATGCTGGGC-3′
	R: 5′-TCCTGGCTGTCCACATGGAC-3′
OCN	F: 5′-CAGACCTAGCAGACACCATGAG-3′
	R: 5′-CGTCCATACTTTCGAGGCAG-3′
Runx2	F: 5′-TTCAACGATCTGAGATTTGTGGG-3′
	R: 5′-GGATGAGGAATGCGCCCTA-3′
GAPDH	F: 5′-TGAAGGTCGGAGTCAACGG-3′
	R: 5′-CTGGAAGATGGTGATGGGATT-3′

*F, forward; R, reverse*.

### Enzyme-Linked Immunosorbent Assay (ELISA) Methods

The ALP, OCN, OPN, and RUNX2 levels in the MC3T3-E1 cell culture supernatants were measured using commercially available ELISA kits (R&D system, USA) in accordance with the manufacturer's protocol. After incubation with primary antibodies against ALP, OCN, OPN, or RUNX2 for 1 h, the optical density (OD) values at 450 nm were obtained using a fluorescence microplate reader.

### Dual-Luciferase Reporter Gene Assays

Oligonucleotides on the 3'UTR of Arhgap11a, Chek2, Dlgap5, and Chek1 mRNAs, containing the corresponding miRNA-binding sites (named Arhgap11a-WT, Chek1-WT, Chek2-WT, and Dlgap5-WT), were artificially synthetized and inserted into the luciferase reporter vector pMIR-reporter (Beijing Huayueyang Ltd., China). Arhgap11a-MUT, Chek1-MUT, Chek2-MUT, and Dlgap5-MUT mutated at the corresponding miRNA-binding sites were also synthetized and inserted these mutant oligonucleotides into the pMIR-reporter. Well-designed pMIR-reporter vectors containing WT or MUT with miRNA mimics were delivered into MC3T3-E1 cells. Firefly and Renilla luciferase activities were measured using the dual-luciferase reporter assay system kit (E1910, Promega, USA), following the instructions provided by the manufacturer, relative to that of renilla luciferase.

### Cell Transfection

The MC3T3-E1 cells undergoing 14-day osteoblast differentiation were treated with miR-211-5p mimic and inhibitor (GenePharma, Shanghai, China) for 48 h using lipofectamine 3,000 reagents (Invitrogen), according to standard protocols, with negative control (NC) of miR-mimic, and -inhibitor served as control.

### Immunoblotting Analysis

The MC3T3-E1 cells were lysed in enhanced radio immunoprecipitation assay (RIPA) with protease inhibitor phenylmethylsulphonyl fluoride (PMSF, Cat. 329-98-6, Amresco, USA). After 10% SDS-PAGE separation and membrane transfer were made, the membrane was incubated with primary Abs for specific Arhgap11a (ab243713, Abcam, Cambridge, UK) and GAPDH (ab181602, Abcam). After incubation with HRP-conjugated secondary antibodies, immunoblots were revealed by chemiluminescence with the ECL kit (Pierce, Thermo Fisher Science, USA) on an Amersham Imager 600 (USA).

### Data Analysis

GraphPad Prism software (version 8.0) was used for data analysis. All measurement data, showing as mean ± standard deviation, are representative of three independent experiments and analyzed by *t*-test, with *p* < 0.05 considered as statistically different.

## Results

### Identification of DGEs Between MC3T3-E1 Cells With and Without Osteoblast Induction

**Figure 1:**
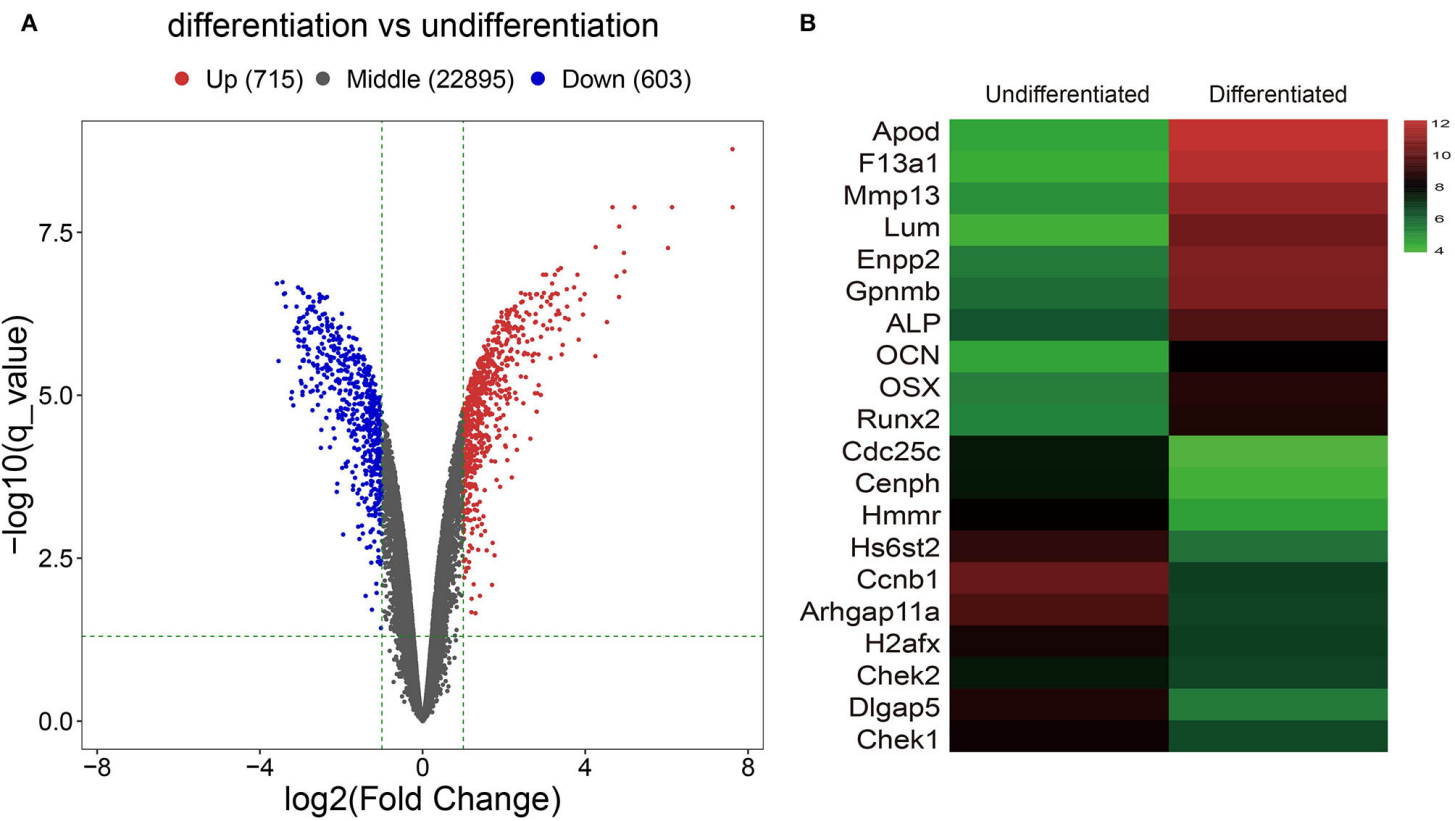
Identification of Differentially expressed genes (DGEs) between MC3T3-E1 cells with and without osteoblast induction. **(A)** The volcano plot; red dots reflect upregulated DGEs (*n* = 715), blue dots reflect downregulated DGEs (*n* = 603), and black dots reflect non-DGEs. **(B)** The heatmap showing expression diversity of 20 representative DGEs in the GSE46400; the color from blue to red shows the expression from low to high.

After analyzing the raw data of the GSE46400 dataset, we identified 715 upregulated DEGs and 603 downregulated DEGs between MC3T3-E1 cells undergoing 14-day osteoblast differentiation and MC3T3-E1 cells without osteoblast induction with more than |log2FC| > 1 (corrected *p* < 0.05) as thresholds, as shown by the volcano plot (Figure 1A). The top 20 DEGs are shown by the heatmap in Figure 1B.

### Functional Enrichment Analyses of DEGs Between MC3T3-E1 Cells With and Without Osteoblast Induction

**Figure 2:**
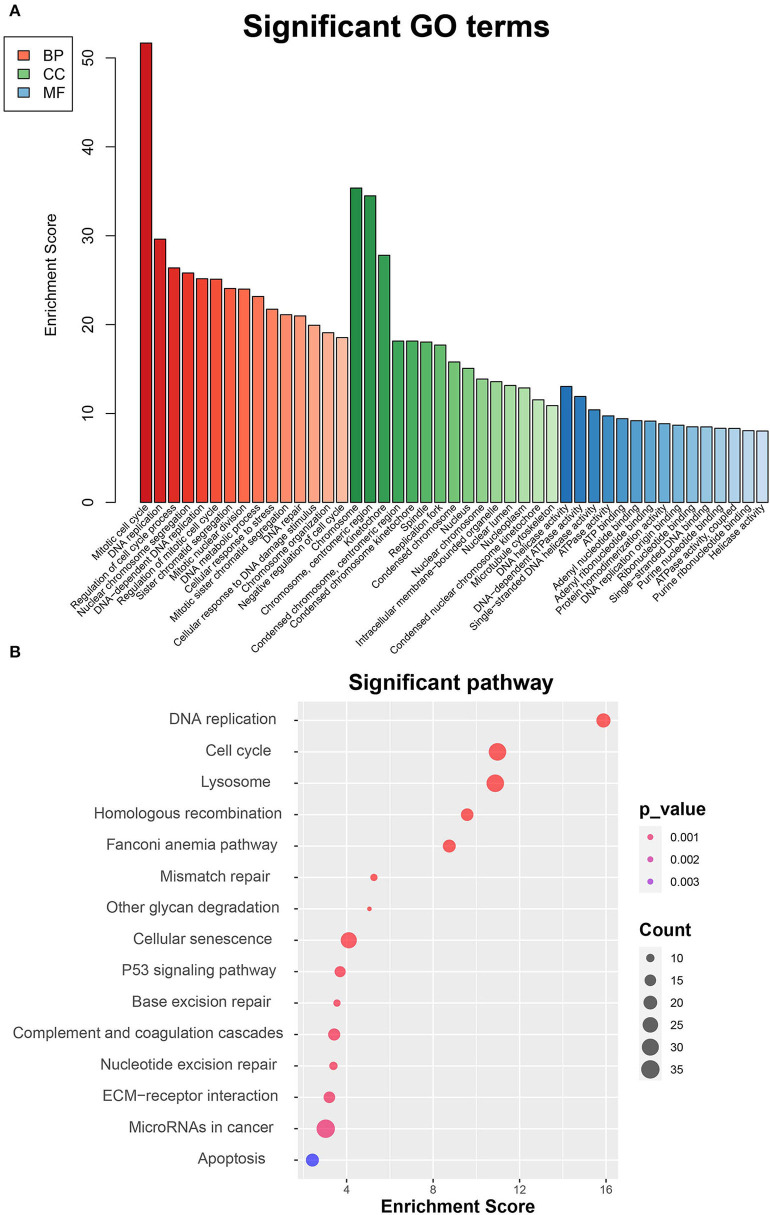
Functional enrichment analyses of DEGs between MC3T3-E1 cells with and without osteoblast induction. **(A)** Significant enrichment of DEGs in the BP, CC, and MF categories (the top 15 GO terms for each category are listed). **(B)** Pathways significantly enriched by the DEGs in the KEGG database; red indicates small *p*-value, and blue indicates large *p*-value; the size of the bubbles indicates the degree of enrichment, and larger bubbles reflect larger generation.

To thoroughly investigate the functional roles of the DEGs between MC3T3-E1 cells undergoing 14-day osteoblast differentiation and MC3T3-E1 cells without osteoblast induction in the GSE46400 dataset, GO enrichment analysis was performed. Those GO terms that were significantly enriched by DEGs are presented in Figure 2A. Based on the BP category, we found that DEGs were significantly enriched in a mitotic cell cycle, DNA replication, and regulation of the cell cycle process. In terms of the CC category, DEGs were mainly enriched in chromosomes, centromeric region, and kinetochore. About the MF category, DEGs were mostly enriched in DNA helicase activity, DNA-dependent ATPase activity, and single-stranded DNA helicase activity. Afterward, DEGs between MC3T3-E1 cells undergoing 14-day osteoblast differentiation and MC3T3-E1 cells without osteoblast induction were mapped into the KEGG database to find out the involvement of the DEGs in most of the known pathways *in vivo*. It was revealed that the DEGs were significantly enriched in DNA replication, a cell cycle, lysosome homologous recombination, and the Fanconi anemia pathway (Figure 2B).

### Identification of miRNA-DEG Regulatory Network

**Figure 3:**
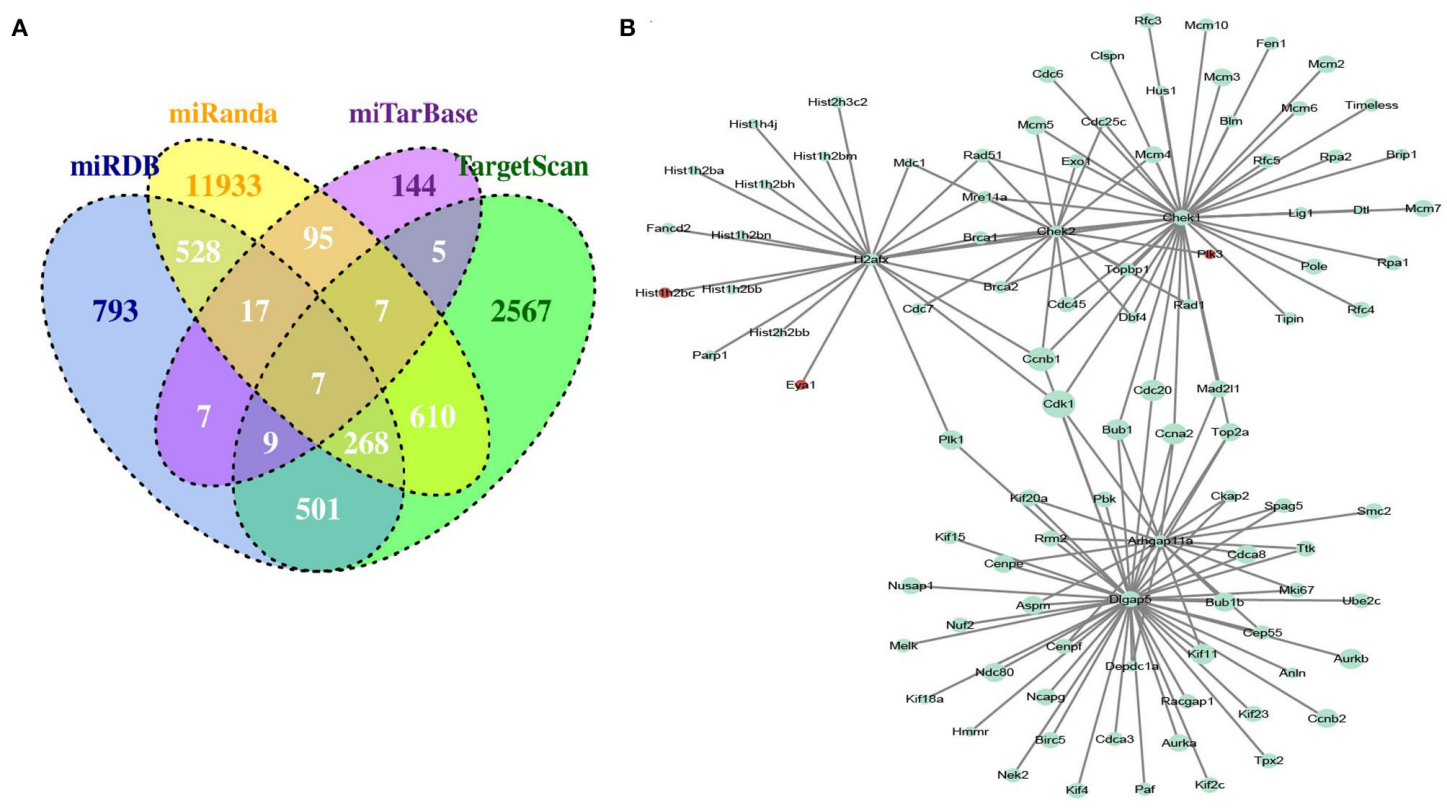
Identification of microRNA (miRNA)-DEG regulatory network during osteogenic differentiation of MC3T3-E1 cells. **(A)** Overlapped putative miRNAs targeting DEGs among the TargetScan, miRTarBase, miRDB, and miRanda databases. **(B)** Construction of protein-protein interaction (PPI) network.

To obtain miRNA-mRNA regulatory networks, we searched the TargetScan, miRTarBase, miRDB, and miRanda databases to find out putative miRNAs targeting DEGs between MC3T3-E1 with and without osteoblast induction. There were 7 overlapped miRNA-mRNA pairs among the four databases, including mmu-miR-204-5p -Arhgap11a, mmu-miR-211-5p-Arhgap11a, mmu-miR-24-3p-H2afx, mmu-miR-3470b-Chek2, mmu-miR-3470b-Dlgap5, mmu-miR-466b-3p-Chek1, and mmu-miR-466c-3p-Chek1 (Figure 3A). Subsequently, we mapped Arhgap11a, H2afx, Chek2, Dlgap5, and Chek1 into the STRING database to construct the PPI network (Figure 3B).

### Candidate miRNA-mRNA Interactions During Osteogenic Differentiation of MC3T3-E1 Cells

**Figure 4:**
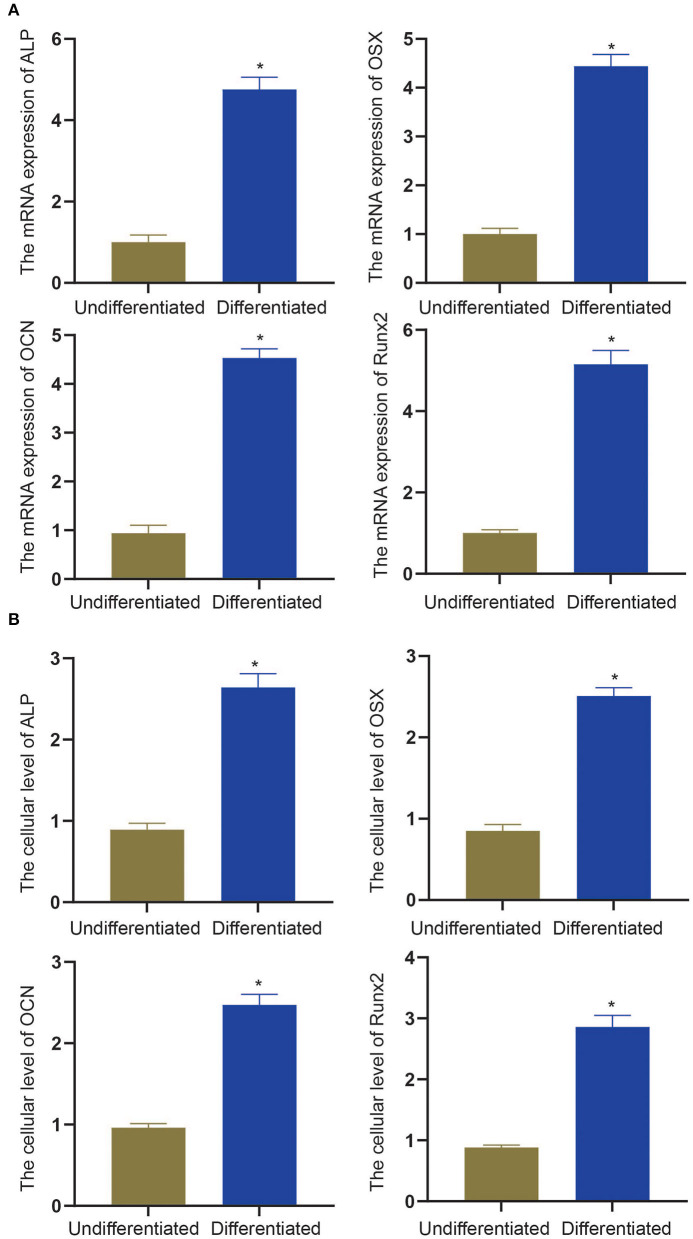
The mRNA expressions and cellular levels of osteoblast markers, ALP, OSX, OCN, and Runx2, were determined by RT-qPCR **(A)** and ELISA methods **(B)** in MC3T3-E1 cells with osteoblast induction and undifferentiated MC3T3-E1 cells. The *p < 0.05 compared with undifferentiated MC3T3-E1 cells. All measurement data, showing as mean ± standard deviation (SD), are representative of three independent experiments and analyzed by *t*-test.

**Figure 5:**
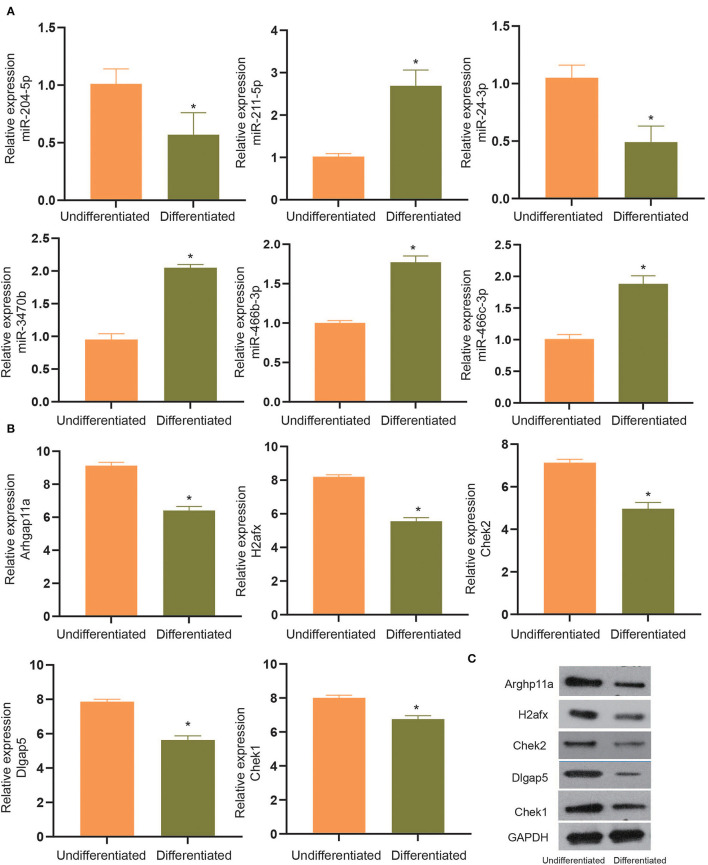
The expressions of miR-204-5p, miR-211-5p, miR-24-3p, miR-3470b, miR-466b-3p, miR-466c-3p **(A)**, mRNA expressions of Arhgap11a, H2afx, Chek2, Dlgap5, and Chek1 **(B)** were determined by RT-qPCR in MC3T3-E1 cells with osteoblast induction and undifferentiated MC3T3-E1 cells. **(C)**, Immunoblots of Arhgap11a, H2afx, Chek2, Dlgap5, and Chek1 proteins, normalized to GADPH, in MC3T3-E1 cells with osteoblast induction and undifferentiated MC3T3-E1 cells. The *p < 0.05 compared with undifferentiated MC3T3-E1 cells. All measurement data, showing as mean ± SD, are representative of three independent experiments and analyzed by *t*-test.

To experimentally verify the results obtained from bioinformatics analysis, MC3T3-E1 cells were cultured with or without the osteogenic differentiation medium for 14 days. The RT-qPCR and ELISA methods were performed to determine the mRNA expressions and cellular levels of osteoblast markers, ALP, OSX, OCN, and Runx2 in the MC3T3-E1 cells (25). It was found that the mRNA expressions and cellular levels of ALP, OSX, OCN, and Runx2 were remarkably elevated in the MC3T3-E1 cells with osteoblast induction compared with undifferentiated MC3T3-E1 cells (*p* < 0.05, Figures 4A,B). Subsequently, we determined the expressions of miR-204-5p, miR-211-5p, miR-24-3p, miR-3470b, miR-466b-3p, miR-466c-3p, mRNA expressions of Arhgap11a, H2afx, Chek2, Dlgap5, and Chek1 in MC3T3-E1 cells cultured with or without the osteogenic differentiation medium. As shown in Figures 5A,B, the expressions of miR-204-5p and miR-24-3p were declined, but the expressions of miR-211-5p, miR-3470b, miR-466b-3p, and miR-466c-3p were increased in the MC3T3-E1 cells with osteoblast induction compared with undifferentiated MC3T3-E1 cells; the mRNA expressions of Arhgap11a, H2afx, Chek2, Dlgap5, and Chek1 were reduced in the MC3T3-E1 cells with osteoblast induction compared with undifferentiated MC3T3-E1 cells (*p* < 0.05). The immunoblotting analysis also demonstrated declines in Arhgap11a, H2afx, Chek2, Dlgap5, and Chek1 protein expressions in the MC3T3-E1 cells with osteoblast induction compared with undifferentiated MC3T3-E1 cells (Figure 5C, *p* < 0.05). These data may suggest the involvement of mmu-miR-211-5p-Arhgap11a, mmu-miR-3470b-Chek2, mmu-miR-3470b-Dlgap5, mmu-miR-466b-3p-Chek1, and mmu-miR-466c-3p-Chek1 during osteogenic differentiation of MC3T3-E1 cells.

### MiR-211-5p Targeting Arhgap11a Was Involved in Osteogenic Differentiation of MC3T3-E1 Cells

**Figure 6:**
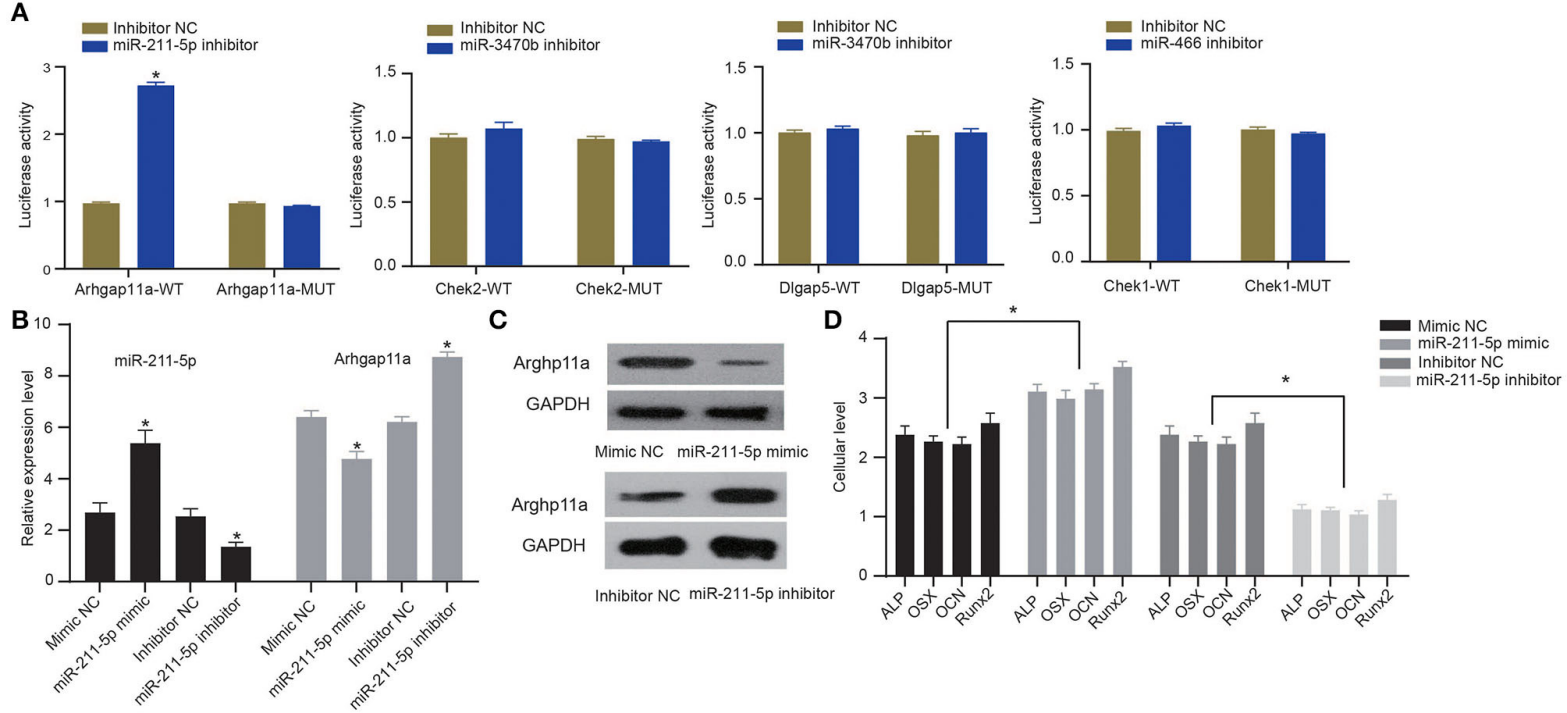
MiR-211-5p targeting Arhgap11a was involved in the osteogenic differentiation of MC3T3-E1 cells. **(A)** Dual-luciferase reporter gene assays were performed to confirm miR-211-5p-Arhgap11a, miR-3470b-Chek2, miR-3470b-Dlgap5, and miR-466-Chek1 interactions. **(B)** RT-qPCR was performed to determine mRNA expressions of miR-211-5p and Arhgap11a in MC3T3-E1 cells undergoing 14-day osteoblast differentiation after miR-211-5p mimic and inhibitor transfections. **(C)** Immunoblots of Arhgap11a, normalized to GADPH, in MC3T3-E1 cells undergoing 14-day osteoblast differentiation after miR-211-5p mimic and inhibitor transfections. **(D)** The cellular levels of osteoblast markers, ALP, OSX, OCN, and Runx2 were determined by ELISA methods in MC3T3-E1 cells undergoing 14-day osteoblast differentiation after miR-211-5p mimic and inhibitor transfections. All measurement data, showing as mean ± standard deviation, are representative of three independent experiments and analyzed by *t*-test. *Means *P* < 0.05.

To verify whether candidate miRNA-mRNA interactions above suggested are involved in osteogenic differentiation of MC3T3-E1 cells, we performed dual-luciferase reporter gene assays to confirm Arhgap11a as the target of miR-211-5p, Chek2, and Dlgap5 as the targets of miR-3470b and Chek1 as the target of miR-466-Chek1 in MC3T3-E1 cells. We only detected a remarkably increased luciferase activity at the promoter of the reporter gene, containing Arhgap11a-WT instead of Arhgap11a-MUT when the miR-211-5p inhibitor was co-transfected, suggesting the presence of miR-211-5p and Arhgap11a interaction in MC3T3-E1 cells (Figure 6A). No evidence of miR-3470b-Chek2, miR-3470b-Dlgap5, and miR-466-Chek1 interactions were observed by dual-luciferase reporter gene assays. Subsequently, we introduced miR-211-5p mimic and inhibitor into MC3T3-E1 cells undergoing 14-day osteoblast differentiation. As shown by RT-qPCR and immunoblotting analysis, declined mRNA and protein expression levels of Arhgap11a were detected in MC3T3-E1 cells with 14-day osteoblast differentiation upon miR-211-5p mimic transfection and elevated mRNA and protein expression levels of Arhgap11a in MC3T3-E1 cells with 14-day osteoblast differentiation upon miR-211-5p inhibitor transfection (Figures 6B,C). Results of ELISA methods (Figure 6D) found increased cellular levels of ALP, OSX, OCN, and Runx2 in MC3T3-E1 cells, with 14-day osteoblast differentiation upon miR-211-5p mimic transfection. An opposite trend was found in these expressions upon miR-211-5p inhibitor transfection. It revealed that miR-211-5p targeting Arhgap11a promoted osteogenic differentiation of MC3T3-E1 cells.

## Discussion

The skeletal system has many functions in the human body, including, but not limited to, supporting body structure and movement, protecting important organs in the body, maintaining mineral balance, and providing bone marrow cells that can differentiate into leukocytes, erythrocytes, and platelets. Various bone diseases, such as osteoporosis, osteoarthritis, metabolic bone dysplasia, and bone cancer, were associated with abnormality of the skeletal system (26, 27). In general, drug therapy is widely used in skeletal system-related diseases. Drug therapy can maintain the relative balance of bone mass in the body through inhibition of bone resorption and promotion of bone formation in patients with bone disease. However, the dosage use of the drug was limited due to adverse reactions in non-skeletal tissues induced by high doses (28). Additionally, non-specific drug therapy must use high-dose systemic administration, which leads to multiple organ injuries and a low response rate (29). Hence, low-toxicity and efficient targeted treatment for skeletal lesions is urgently required.

In certain conditions, stem cells can differentiate into different cells, such as osteoblasts, nerve cells, cardiomyocytes, chondrocytes, and adipocytes. Osteoblast differentiation consists of cell proliferation, extracellular matrix formation and maturation, and bone matrix mineralization. Cells at each stage show different characteristics, and gene expression related to osteogenesis is also different at each stage. MiRNAs, as non-coding RNA, are considered to be one of the most effective regulators of gene expression at the post-transcriptional level. It has been reported that various miRNAs regulated Runx2 expression and participated in the process of osteogenesis (30). In the present study, putative miRNA-mRNA pairs were initially revealed to be involved in the process of osteogenic differentiation in MC3T3-E1 cells by microarray data analysis. Our *in vivo* data showed the expressions of miR-204-5p and miR-24-3p were declined, the expressions of miR-211-5p, miR-3470b, miR-466b-3p, and miR-466c-3p were increased, the mRNA expressions of Arhgap11a, H2afx, Chek2, Dlgap5, and Chek1 were reduced in the MC3T3-E1 cells with osteoblast induction compared with undifferentiated MC3T3-E1 cells, although existing evidence showed the contributory role of miR-204-5p during osteogenic differentiation. For example, Yu et al. found that, in calcific aortic valve disease, increased expression of miR-204-5p inhibited Runx2 expression at the post-transcriptional level, resulting in suppression of osteogenic differentiation finally (31). Another study on ankylosing spondylitis presented by Zhao et al. also indicated decreased miR-204-5p expression and increased activity of ALP, as well as elevated expression of RUNX2 and OCN, contributed to enhancing osteogenic differentiation of ankylosing spondylitis fibroblasts (32). Wu et al. demonstrated lncRNA LEF1-AS1 favored osteogenic differentiation of dental pulp stem cells *via* repressing the expression of miR-24-3p (33). Another research also indicated miR-24-3p overexpression inhibited mRNA and protein expressions of OCN, OPN, and ALP (34). However, considering miRNAs work by reducing mRNA levels, we removed two pairs: miR-204-5p-Arhgap11a and miR-24-3p-H2afx, out of the hypothesis of candidate miRNA-mRNA pairs involved in MC3T3-E1 osteogenic differentiation. The H2afx, also known as H2ax, has been proven to be a marker to distinguish patients with or without liver and lung fibrosis (35) and marrow fibrosis (36). Previous studies attached more attention to the oncogenic role in cancers, including basal-like breast cancer subtype (37) and lung adenocarcinoma (38). The Chek2 plays a key role in cell response to DNA damage, and Chek2 loss has been confirmed to improve partially skeletal growth in parathyroid hormone-related peptide 1-84 knockin mice (39). It was reported Dlgap5 was involved in cell proliferation, differentiation, and migration in oral squamous cell carcinoma (40). Its upregulation was associated with worse overall survival in colorectal cancer (41). Previous evidence increased expression of Chek1-induced cell proliferation of multiple myeloma, leading to a poor prognosis (42). Researchers Liu et al. also revealed, in bone metabolism, Chek1 inhibitors suppressed the development of bone resorption induced by osteoclasts (43). Finally, we hypothesized candidate miRNA-mRNA pairs among mmu-miR-211-5p-Arhgap11a, mmu-miR-3470b-Chek2, mmu-miR-3470b-Dlgap5, mmu-miR-466b-3p-Chek1, and mmu-miR-466c-3p-Chek1 involved in MC3T3-E1 osteogenic differentiation.

Candidate miRNA-mRNA interactions above suggested were verified by dual-luciferase reporter gene assays in osteogenic differentiation of MC3T3-E1 cells. We only observed evidence of miR-211-5p and Arhgap11a interaction in MC3T3-E1 cells. This study revealed miR-211-5p was upregulated, and Arhgap11a was downregulated in MC3T3-E1 cells after osteogenic differentiation induction. The following functional studies indicated that the miR-211-5p targeting Arhgap11a possibly promotes osteogenic differentiation of MC3T3-E1 cells, which conformed to their correlation revealed in the GSE46400 dataset. The MiR-211-5p is a member of the miR-211 family. A previous study confirmed miR-211 facilitated MSC migration and improved the therapeutic efficacy of MSCs (44). In the study reported by Wang et al., miR-211-5p promoted osteogenic differentiation by regulating the DUSP6-mediated ERK/SMAD/β-catenin pathway (45). Likewise, miR-211-5p is reduced in articular cartilage tissues in an osteoarthritis rat model, but it is upregulated during chondrocyte differentiation of ATDC5 cells, repressing the expression of pro-inflammatory cytokines and proteinases responsible for cartilage matrix degradation (46). The Arhgap11a is a protein-coding gene that locates on chr15q13.3, encoding a member of GTPase-activating proteins (RhoGAPs), which are upstream regulators of Rho GTPases. The Rho GTPases are a subfamily of the Ras superfamily proteins, which play central roles in multiple biological processes, such as cell motility, cell polarity, cell cycle progression, cell adhesion, migration, and invasion (47). Our study firstly demonstrated targeted inhibition of Arhgap11a by miR-211-facilitated osteogenic differentiation of MC3T3-E1 cells.

This study firstly reveals the involvement of candidate miRNA-mRNA pairs in the osteogenic differentiation of MC3T3-E1 cells using an *in silico* approach. The following *in vitro* data support the involvement of miR-211-5p and Arhgap11a interaction during osteogenic differentiation of MC3T3-E1 cells. The MiR-211-5p may promote osteogenic differentiation of MC3T3-E1 cells by targeted inhibition of Arhgap11a. However, RNA sequencing on saos-2 or hfob1.19 cells between 14-day osteoblast differentiation and control will be performed in further investigations, and more data are needed to verify the involvement of signaling pathways in the process of osteoblast differentiation, in a bid to find out novel targets to promote the osteogenesis process during bone repair.

## Data Availability Statement

The original contributions presented in the study are included in the article/supplementary material, further inquiries can be directed to the corresponding author.

## Author Contributions

WWJ conceived the study, wrote the initial draft, and edited the final version. GFZ prepared the experimental resources and the software. XZ and JTW performed data analysis and interpretation. TW contributed to charts. HFL assisted in manuscript draft and revision. All authors contributed significantly to the initiation and design of the study and manuscript approval.

## Funding

This study received support from the Basic Scientific Research Operational Expenses Scientific Research Projects of Heilongjiang Provincial Colleges and Universities (2019-KYYWF-1239).

## Conflict of Interest

The authors declare that the research was conducted in the absence of any commercial or financial relationships that could be construed as a potential conflict of interest.

## Publisher's Note

All claims expressed in this article are solely those of the authors and do not necessarily represent those of their affiliated organizations, or those of the publisher, the editors and the reviewers. Any product that may be evaluated in this article, or claim that may be made by its manufacturer, is not guaranteed or endorsed by the publisher.
